# Microevolution of Mycobacterium tuberculosis Subpopulations and Heteroresistance in a Patient Receiving 27 Years of Tuberculosis Treatment in Germany

**DOI:** 10.1128/AAC.02520-20

**Published:** 2021-06-17

**Authors:** Lindsay Sonnenkalb, Gerald Strohe, Viola Dreyer, Sönke Andres, Doris Hillemann, Florian P. Maurer, Stefan Niemann, Matthias Merker

**Affiliations:** a Molecular and Experimental Mycobacteriology, Research Centre Borstel, Borstel, Germany; b Landratsamt Karlsruhe, Gesundheitsamt, Karlsruhe, Germany; c National and Supranational Reference Centre for Mycobacteria, Research Centre Borstel, Borstel, Germany; d Institute of Medical Microbiology, Virology and Hygiene, University Medical Centre Hamburg-Eppendorf, Hamburg, Germany; e German Centre for Infection Research (DZIF), Partner site Hamburg-Lübeck-Borstel, Borstel, Germany

**Keywords:** *Mycobacterium tuberculosis*, antibiotic resistance, antibiotics, evolution, microevolution, multidrug resistance, patient treatment, relapse, treatment failure, tuberculosis

## Abstract

Preexisting and newly emerging resistant pathogen subpopulations (heteroresistance) are potential risk factors for treatment failure of multi/extensively drug resistant (MDR/XDR) tuberculosis (TB). Intrapatient evolutionary dynamics of Mycobacterium tuberculosis complex (Mtbc) strains and their implications on treatment outcomes are still not completely understood. To elucidate how Mtbc strains escape therapy, we analyzed 13 serial isolates from a German patient by whole-genome sequencing. Sequencing data were compared with phenotypic drug susceptibility profiles and the patient’s collective 27-year treatment history to further elucidate factors fostering intrapatient resistance evolution. The patient endured five distinct TB episodes, ending in resistance to 16 drugs and a nearly untreatable XDR-TB infection. The first isolate obtained, during the patient’s 5th TB episode, presented fixed resistance mutations to 7 anti-TB drugs, including isoniazid, rifampin, streptomycin, pyrazinamide, prothionamide, para-aminosalicylic acid, and cycloserine-terizidone. Over the next 13 years, a dynamic evolution with coexisting, heterogeneous subpopulations was observed in 6 out of 13 sequential bacterial isolates. The emergence of drug-resistant subpopulations coincided with frequent changes in treatment regimens, which often included two or fewer active compounds. This evolutionary arms race between competing subpopulations ultimately resulted in the fixation of a single XDR variant. Our data demonstrate the complex intrapatient microevolution of Mtbc subpopulations during failing MDR/XDR-TB treatment. Designing effective treatment regimens based on rapid detection of (hetero) resistance is key to avoid resistance development and treatment failure.

## INTRODUCTION

With an estimated 10 million new cases in 2018 and half a million new multidrug-resistant (MDR) cases, tuberculosis (TB) caused by bacteria of the Mycobacterium tuberculosis complex (Mtbc) continues to be the most devastating disease caused by a single infectious agent (1). Due to transmission and treatment failures, MDR-TB (defined as resistance to isoniazid [H] and rifampicin [R]) and extensively drug resistant (XDR) TB (includes additional resistance to a fluoroquinolone [FQ] and second-line injectable drug) cases continue to rise. Worldwide, TB treatment success rates are about 82% for susceptible infections, decreasing to as low as 55% in MDR-TB cases (1). In approximately 90% of failed treatment cases, relapse occurs within 12 months of completed treatment (2, 3). Not all possible causes of treatment failure are well defined, but patient noncompliance and inappropriate drug regimens are most important (4, 5). Additionally, patient metabolism, pharmacokinetics, and pharmacodynamics also play a role in resistance acquisition and treatment failure (6). In efforts to overcome these treatment limitations, strategies such as drug administration programs, centralized treatment methods, comprehensive and rapid drug susceptibility testing, and precision medicine have been implemented (7, 8).

A number of studies, which have utilized sequencing techniques on serial isolates from the same patient, have found that resistance variants arise and are selected in failing treatment regimens from heterogeneous populations (9–14). These heterogeneous populations can comprise of several resistant subpopulations, also known as heteroresistance. Continued drug exposure on these populations ultimately selects and fixes a single resistance-mediating mutation. *In vitro* studies have further demonstrated that individual mutations can lead to a significant (and variable) reduction or increase in bacterial fitness, which likely explains the selection and loss of certain mutations during therapy (15, 16).

Understanding the mode and conditions under which drug resistance-associated mutations arise and are selected is paramount when considering diagnostic procedures and treatment regimens. For this understanding, we need rapid and sensitive diagnostics like next-generation sequencing (NGS) amplicon sequencing, which allows for the detection of genotypic drug resistance and heteroresistance populations in patient sputum samples at low frequencies (17). Such tools could guide better treatment regimens, as they have been shown to detect resistance and diverse populations as well as, or better than, phenotypic drug susceptibility testing (pDST) (18, 19).

In this study, we investigated intrapatient Mtbc microevolution within a single patient who suffered 5 distinct TB episodes resulting in a TB treatment of 27 years. We aligned whole-genome sequencing (WGS) data of 13 serial isolates collected during the final infectious episode, with pDST data and the patient’s treatment history, to explore the connection between treatment regimens and evolutionary dynamics.

## RESULTS

### Case history.

The patient, of German descent, was first diagnosed with pulmonary TB in Western Germany in the late 1950s at 4 years old. After 4 months of treatment with H, para-aminosalicylic acid (PAS), and dihydrothenat injectable (a derivative of streptomycin), therapy was concluded and the patient was considered cured. Over 39 years, the patient endured an additional 4 relapse events, with treatment lasting 4 months, 41 months, 88 months, and finally 216 months until the patient died of the infection (see Table S1 in the supplemental material). It is not clear whether each episode was attributed to reinfection, reactivation, or both, as only bacterial isolates from the last TB episode were recovered.

The patient disclosed treatment noncompliance to clinicians, stated as “not properly administering his medication” during some previous treatment periods. Patient records did not indicate that an HIV coinfection was present; however, immunosuppressive activity, such as alcohol abuse and smoking cigarettes, was noted (20, 21).

**FIG 1:**
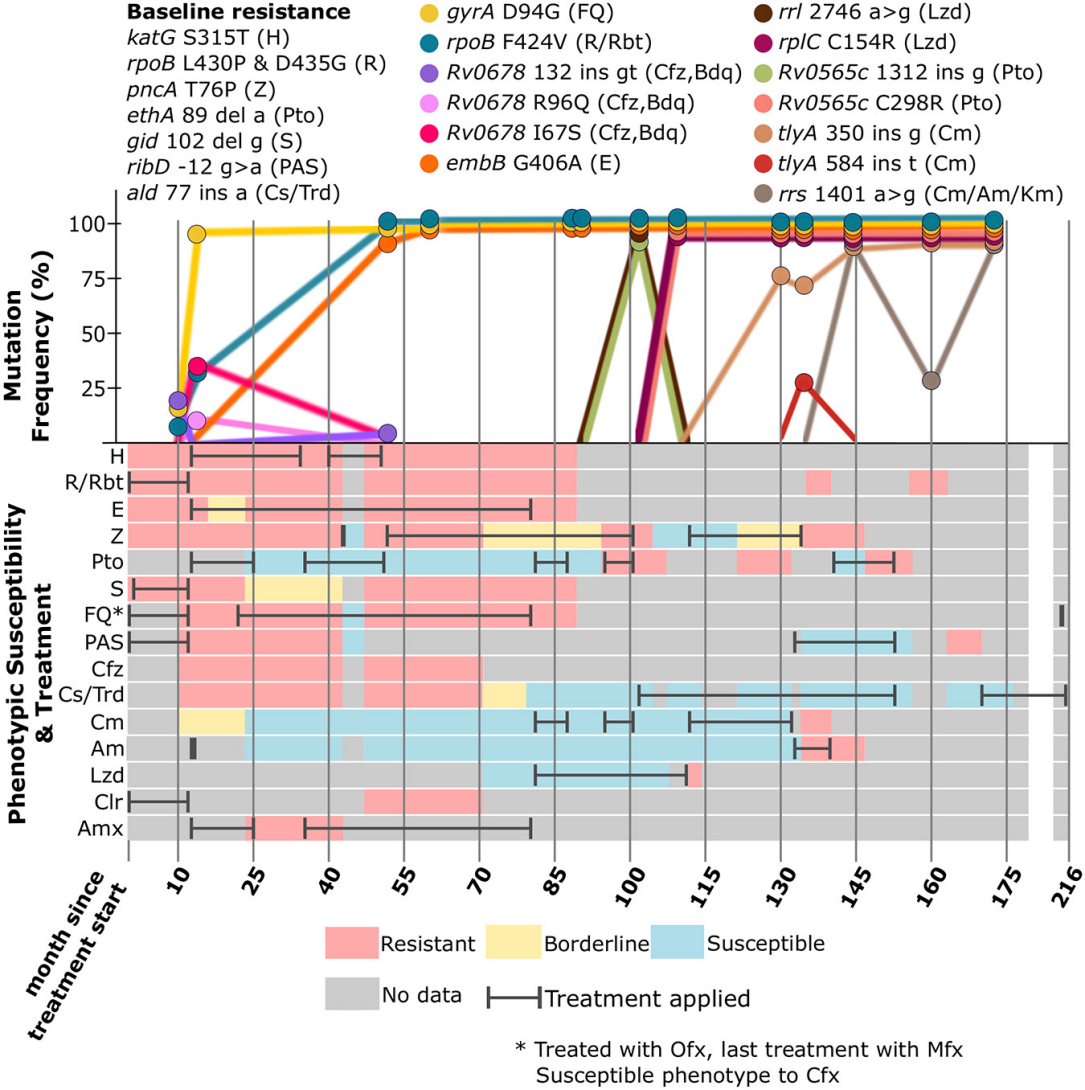
Patient treatment history, bacterial phenotypic drug susceptibility test results, and acquisition of resistance-mediating mutations. Newly emerging mutations implicated in resistance are color coded in the top panel. Mutation frequency (*y* axis) is inferred from next-generation sequencing (NGS) data, i.e., frequency of the resistance allele, and time points available for NGS analysis are indicated by circles. Lines represent changes of mutation frequencies over time. Phenotypic drug susceptibility test (pDST) results are color coded in the bottom panel. Horizontal bars indicate application of a drug (overlaying the pDST). Both pDST and drug regimens are based on the patient record. Recent guidelines for drug susceptibility testing of M. tuberculosis complex isolates do not support the critical test concentration for the antibiotics Clr, Amx, Cs/Trd, and PAS. Am, amikacin; Amx, amoxicillin+clavulanic acid; Bdq, bedaquiline; Cfx, ciprofloxacin; Cfz, clofazimine; Clr, clarithromycin; Cm, capreomycin; Cs, cycloserine; E, ethambutol; FQ, fluoroquinolone; H, isoniazid; Km, kanamycin; Lzd, linezolid; Mfx, moxifloxacin; OP, outpatient; PAS, para-aminosalicylic acid; Pto, prothionamide; R, rifampicin; Rbt, rifabutin; S, streptomycin; Trd, terizidone; Z, pyrazinamide.

Initial pDST was conducted during the patient’s 4th TB episode that revealed the Mtbc strain was already resistant to five drugs. Several months later, additional testing confirmed MDR-TB. Over the final 18-year treatment course, the antibiotic regimen included a maximum of 3 active drugs but most often 2 or 1 (Table S1; Fig. 1). During this final TB episode, sequential bacterial isolates were recovered spanning 13 years of the treatment period. WGS revealed initial resistance to seven drugs (nine when considering low frequency populations) and indicated the acquisition of resistance to five additional drugs over this time. Although pDST designated similar resistance profiles as the genotype predicted, inconsistent results for some antibiotics were recurrent, alternating between resistant and susceptible.

### Preexisting and treatment-selected resistance-mediating mutations.

**FIG 2:**
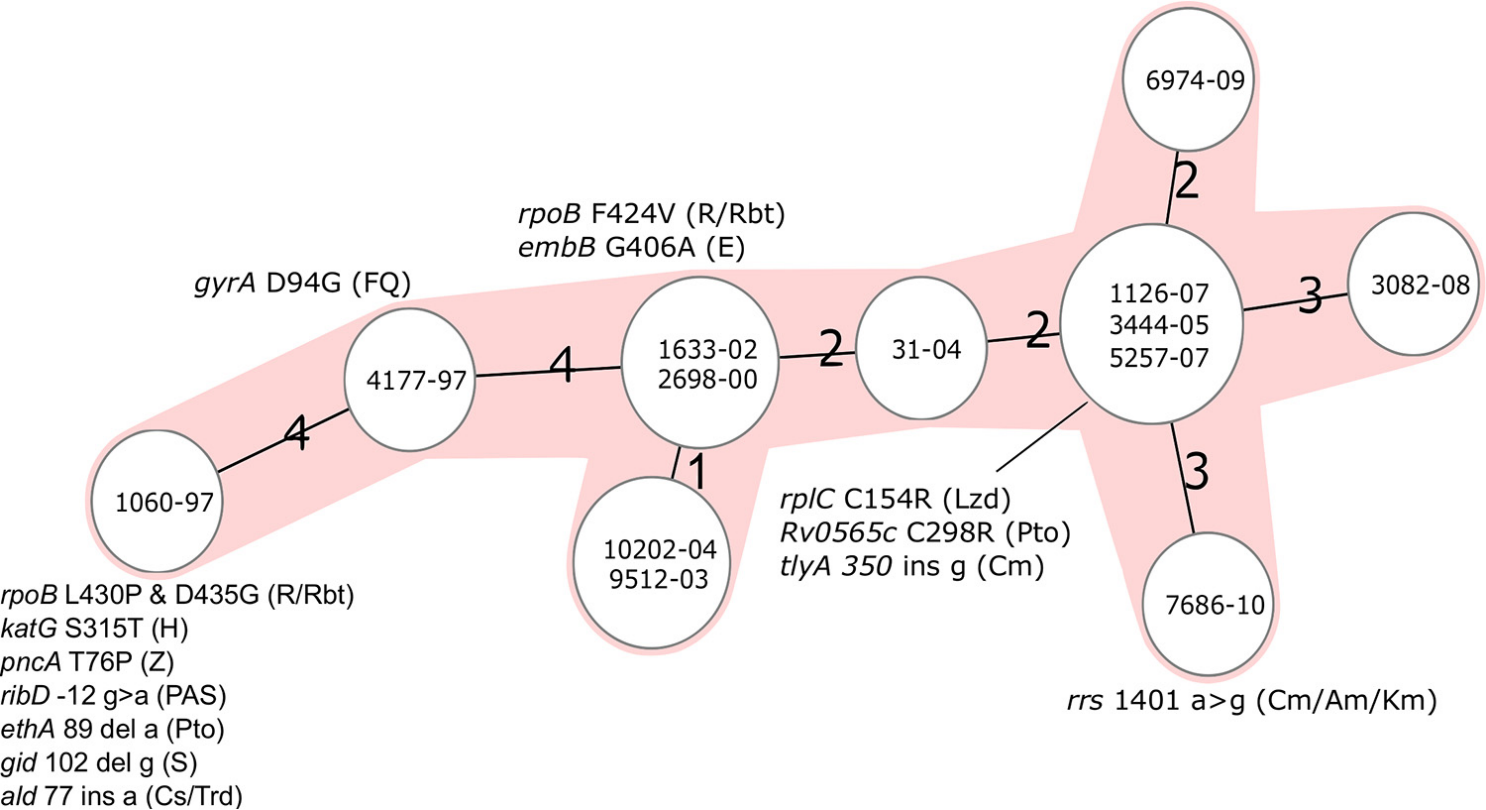
Intrapatient microevolution of a multidrug resistant (MDR) Mycobacterium tuberculosis complex (Mtbc) strain. Minimum spanning tree based on a core genome multilocus sequence type (cgMLST) analysis of 13 serial patient isolates. The number of allele differences are indicated on connecting lines. Resistance- mediating mutation (and associated drug) are noted next to the isolate in which the mutation became fixed in the genome. Am, amikacin; Cm, capreomycin; Cs, cycloserine; E, ethambutol; FQ, fluoroquinolone; H, isoniazid; Km, kanamycin; Lzd, linezolid; PAS, para-aminosalicylic acid; Pto, prothionamide; R, rifampicin; Rbt, rifabutin; S, streptomycin; Z, pyrazinamide.

Overall, WGS analysis was performed on 13 serial isolates to predict drug resistance and drug susceptibility (termed genotypic drug susceptibility testing [gDST]) (Fig. 1; see Table S2 in the supplemental material). All bacterial isolates obtained belonged to Mtbc lineage 4.7 and had a maximum distance between sequential isolates of less than five alleles, while showing a strictly clonal evolution, thus excluding a reinfection (Fig. 2).

The first isolate available for WGS was collected 10 months after beginning treatment, which revealed resistance-mediating mutations to 7 different antibiotics, i.e., *katG* S315T for H, *rpoB* L430P in combination with D435G for R, *pncA* T76P for pyrazinamide (Z), *ribD* at −12 g > a for para-aminosalicyclic acid (PAS), *ethA* 89 del a for prothionamide (Pto), *gidB* 102 del g for streptomycin (S), and *ald* 77 ins a for cycloserine-terizidone (Cs/Trd) (where del is deletion and ins is insertion). Antibiotics which had been included in previous regimens were H, R, ethambutol (E), Z, S, PAS, capreomycin (Cm), clarithromycin (Clr), Pto, and Trd for a minimum of 3 consecutive months, although most were applied for over 10 months (Table S1).

The isolate analyzed from month 13 indicated fluoroquinolone (FQ) resistance, mediated by the mutation *gyrA* D94G, after about 1 year of ofloxacin (Ofx) treatment. No further isolate was collected until month 47, which then revealed resistance to E mediated by the mutation *embB* G406A. However, phenotypic resistance to E was detected already in month 22, while E was included in the treatment since month 13. Additionally, a third R-associated mutation (*rpoB* F424V) emerged, while R was still included in the treatment regimen (Fig. 1).

In month 118, after 26 months of linezolid (Lzd) treatment, the resistance-mediating mutation *rplC* C154R emerged to fixation. From the isolate obtained in month 130, we identified the mutation *tlyA* 350 ins g likely mediating resistance against the second-line injectable drug Cm, observed after 18 consecutive months of Cm treatment. Additionally, in month 144, after 8 months of amikacin (Am) treatment, the mutation *rrs* 1401 a > g arose conferring cross-resistance against all second-line injectable drugs (Cm, Am, and kanamycin [Km]).

### Insufficient treatment regimens.

The treatment of this patient was highly complex, with regimens frequently changing. However, the treatment regimens were not always congruent with pDST results (Fig. 1). This resulted in suboptimal regimens consisting of fewer than 3 active drugs for virtually the last 18 years of treatment. Moreover, only two, one, or even no active drugs were administered at several time points (Table S1; Fig. 1).

In the final TB treatment episode, when considering only gDST, we observed only one instance in which a maximum of three active drugs (E, Am, and amoxicillin+clavulanic acid [Amx]) were included in treatment, in months 13 and 14. On the other hand, Amx may not be considered, as it is not typically prescribed for TB, but it has presented synergistic affects during MDR-TB treatment (22, 23). From month 1 through 12, Clr appeared to be the only active drug applied but has since shown to be trivial for TB treatment due to intrinsic resistance by Mtbc strains (24, 25). However, a study in 1996 indicated that Clr might have a synergistic affect when included in combination treatments with R, H, and/or E *in vitro* (26).

In most instances when new resistance arose, only 1 or 2 active drugs were prescribed; this was notably seen with Lzd (Table S1). From month 88 through 94, Lzd was the only active drug included in treatment, and the patient gained Lzd resistance by the fixation of mutation *rrl* 2746 a > g in month 104 of treatment, followed by *rplC* C154R in month 108, when the isolate was still indicated as phenotypically susceptible (Fig. 1).

### Heteroresistance and microevolution.

To resolve discrepancies between pDST and gDST and to investigate the evolutionary trajectories of emerging resistant subpopulations, i.e., heteroresistance, we investigated the presence of mutations down to a frequency of 1% and included only statistically verified variants in our report (Table S2; see Table S3 in the supplemental material). Overall, we found 10 instances of low frequency resistance-mediating mutations (below 75% frequency), which either emerged months before reaching fixation, fluctuated at different frequencies over the course of the therapy, and/or disappeared from the bacterial population (Fig. 1; Table S2).

For instance, FQ resistance mediated by *gyrA* D94G was already detected in month 10 at a frequency of 21%. Also, a third *rpoB* F424V mutation (with unclear phenotypic effect) could be observed in month 10 at 5% and month 13 at 30%, and it reached fixation in the subsequent isolate collected in month 47 at 96% frequency.

Heteroresistance could also be observed with regard to Cm (Fig. 1; Table S2). In month 130, one *tlyA* insertion was detected, namely, 350 ins g at 79% frequency, 18 months after Cm was introduced into the treatment regimen. In the subsequent isolate collected 5 months later, the additional frameshift mutation *tlyA* 584 ins t was detected at 28% and the 350 ins g mutation decreased slightly to 71%. Finally, 9 months later, *tlyA* 350 ins g reached 97% frequency, while the second subpopulation *tlyA* 584 ins t was not again detected.

Cross-resistance to all second-line injectable drugs mediated by *rrs* 1401 a > g was then found at 100% frequency in isolate 3082-08 collected in month 144, decreased to 29% frequency in the subsequent isolate, and then increased again to 99% thereafter.

In the first collected isolates, we discovered a low frequency mutation (*Rv0678* 132 ins gt at 22%) potentially conferring cross-resistance to clofazimine (Cfz) and/or bedaquiline (Bdq). This frameshift mutation was lost in the following isolate, correlating with the emerging mutations *Rv0678* I67S at 36% and *Rv0678* R96Q at 10% frequencies. Both mutations correlated with Cfz resistance pDST, although there is no mention of Cfz inclusion in the drug regimens throughout all the patient’s treatments. Again, *Rv0678* 132 ins gt was detected in month 47 at 3.6%. All *Rv0678* mutations eventually disappeared from the population by month 71 of treatment.

### Putative compensatory and tolerance effects.

Finally, two high frequency mutations without a direct effect on resistance were detected. A mutation in the gene *prpR* (*Rv1129c*) F334L involved in drug tolerance (27) was first found in month 92, was not observed in month 104 isolate, but was then fixed in the population after month 108 (Table S2).

Two mutations arose in monooxygenase *Rv0565c*, a gene with a possible compensatory mechanism which overcomes fitness defects brought on by mutations in the monooxygenase *ethA* gene (activating the drugs ethionamide [Eto] and Pto) (28). These mutations in *Rv0565c* developed in isolates after the mutation *ethA* 89 del a. First the mutation *Rv0565c* 1312 ins g arose to 97% frequency in month 104 but was lost in all following isolates. In the subsequent isolate from month 108, a second mutation *Rv0565c* C298R was detected at 99% and remained fixed in the population.

## DISCUSSION

Our analysis of Mtbc microevolution over the complex treatment of one patient revealed that fixation of resistance mutations in the bacterial population is a dynamic process. The emergence and extinction of different subpopulations are likely triggered by therapy changes and suboptimal treatment regimens. The rapid detection of heteroresistance by NGS techniques could offer new opportunities for intervention measures and a more effective treatment regimen.

Through the application of WGS, we could show that resistance evolution was influenced by long periods of ineffective treatment regimens, with several periods of only one or two active drugs applied. Suboptimal therapy design was partially due to minimal options of active drugs, especially in the last 18 years of treatment. In fact, there were several time points in which drugs were still applied, despite resistant pDST. For example, between month 48 and 81, the patient was treated with E, Ofx, Z, and Amx, despite already presenting previous phenotypic resistant to E, Ofx, and Z. Genotypic tests were not performed at that time, but WGS analysis retrospectively confirmed resistance to E (*embB* G406A), Ofx (*gyrA* D94G), and Z (*pncA* T76P). Finally, frequent regimen changes and poor treatment design may have been fostered by changes in clinic (treated in 12 clinics within Germany throughout the patient’s life), several laboratories reporting pDST results, and also lack of remaining effective drugs.

During our longitudinal analysis, we observed at several time points heterogeneous subpopulations, indicating emerging resistance during the treatment course. As multiple resistant subpopulations can arise and coexist during MDR-TB treatment, diagnostic approaches need to be employed, such as amplicon sequencing of sputum, which enables the rapid and sensitive detection of resistance and low frequency resistance subpopulations (29). Such information could then be used to rapidly change treatment regimens and help avoid treatment failure. In order to enact this approach in the future, one also needs to consider bacterial subpopulations can reside at different sites of infection, each following their own microevolution (30). In order to rapidly respond to changes of mutation frequencies, sampling should be performed regularly to capture the entire intrapatient strain diversity.

The limitation of this study is the retrospective character and that most of the patient isolates were not available to repeat pDST according to current standards. Of note, any cultivation step prior to pDST and DNA isolation for NGS can potentially influence the mutation frequencies reported in our study. Furthermore, individual sputum specimens may only comprise a fraction of the overall Mtbc diversity within the patient. Additionally, distant anatomical lesions can contain different bacterial populations with distinct resistance mutation profiles (31). As mentioned, patient records were compiled from 12 different clinics and external pDST results were sometimes contradicting the data acquired at National Reference Centre Borstel, but as presented, they should represent the information given to the attending clinician at that time. Rationales for the design of individual therapy episodes could not be retrieved.

Additionally, intrapatient competition of subpopulations may also induce the emergence of putative compensatory and tolerance mutations described as *Rv0565c* C298R, *Rv0565c* 1312 ins g (Pto), and *prpR* F334L (27, 28). Drug tolerance-associated mutations and compensatory mutations are discussed for their potential clinical relevance, as they may affect bacterial growth and mutation rates. These types of mutations and mechanisms are generally not detected in typical pDST. In the case for the emergence of *Rv0565c* mutations, in an *ethA*-deficient genetic background, and coinciding with a Pto-resistant phenotype, we cannot exclude that *Rv0565c* is also implicated in resistance against Pto itself.

We demonstrated that resistance development in a failing MDR-TB therapy involved an arms race of coexisting bacterial subpopulations. Continued treatment with less than four active drugs likely selected for the most resistant clone over time. This study also highlights the benefits of genomic resistance testing, which can improve treatment with drugs lacking recommendations of critical test concentrations (PAS and Cs) or drugs with poorly reproducibly pDST, such as Z and E (32, 33).

## MATERIALS AND METHODS

### Phenotyping and DNA isolation of serial patient isolates.

Sputum samples were collected from a single patient in Germany and were received by the National Reference Centre of Research Centre Borstel Leibniz Lung Centre (NRC-Borstel). The bacteria recovered from the sputum samples were stored at NRC-Borstel and were regrown on Löwenstein-Jensen slants for DNA isolation for this study. The bacteria were cultured at 37°C until colonies were visible (about 3 weeks); and colonies were then scraped from the medium using a sterile loop, transferred to 400-μl Tris-EDTA (TE) buffer, and heat killed in an 80°C water bath for 20 minutes. Isolation of genomic DNA was conducted using the standard cetyltrimethylammonium bromide (CTAB) method as described previously (34). Drug susceptibility testing conducted at NRC-Borstel used Mycobacterium growth indicator tubes (MGIT; Becton, Dickinson Microbiology Systems, Sparks, MD) or agar dilution on Middlebrook 7H10 (or 7H11) plates with the recommended critical concentrations at the time of pDST; pDST methods conducted by other labs could not be retrieved. Patient treatment history was collected and provided by the Rastatt Department of Health and the state health office of Stuttgart in Germany. The pDST results presented were also compiled by the Rastatt Department of Health. Although there are not clearly defined critical concentrations for Clr and Amx, these drugs were included in pDST in the patient treatment history; therefore, we presented the resistance profile for these drugs as it was documented.

### Next-generation sequencing and resistance prediction of serial patient isolates.

Genomic DNA was sequenced using Illumina NextSeq 500 technology and Nextera XT library preparation kits according to manufacturer’s guidelines. Mutations (single-nucleotide polymorphisms, small insertions, and deletions) were detected with the MTBSeq pipeline adjusting the minimum coverage (of two reads) to distinguish mutations at low frequencies (35). Variant calls were first filtered in the MTBSeq low frequency output with a threshold of at least 1 read in both forward and reverse orientation and a minimum of 1% frequency. All variant calls with a frequency below 75% were statistically verified using the binoSNP variant detection tool and calculated by number of calls with a minimum phred base quality of 20 (36). All low frequency variants with a *P* value of ≤0.05 were included (Table S3). Genes in highly repetitive regions, e.g., proline-glutamate (PE), proline-proline-glutamate (PPE) genes, and genes with polymorphic-GC-rich sequences (PGRS), were not considered. For the genotypic prediction of drug resistance and drug susceptibility, mutations in 92 genes implicated in resistance to 21 different anti-TB drugs were screened; phylogenetically informative mutations were not considered (37). In the absence of a known resistance mutation, the isolate was considered drug susceptible.

Of note, false-negative results could not be excluded, as many of the historic specimens were no longer available for repeated pDST. A minimum spanning tree was calculated with a core genome multilocus sequence type (cgMLST) approach with SeqSphere v5.9 (Ridom, Münster, Germany) as described previously and by pairwise ignoring missing values (38).

### Data availability.

The bacterial DNA sequencing data supporting the conclusion of the manuscript are available at the European Nucleotide Archive (https://www.ebi.ac.uk/ena/browser/home). Accession numbers are ERS4932039 (patient isolate identification no. 1060-97), ERS4932040 (4177-97), ERS4932041 (2698-00), ERS4932042 (1633-02), ERS4932043 (9512-03), ERS4932044 (31-04), ERS4932045 (10202-04), ERS4932046 (3444-05), ERS4932047 (1126-07), ERS4932048 (5257-07), ERS4932049 (3082-08), ERS4932050 (6974-09), and ERS4932051 (7686-10).
