# Supplementary material for: Microevolution of Mycobacterium tuberculosis Subpopulations and Heteroresistance in a Patient Receiving 27 Years of Tuberculosis Treatment in Germany
Source: Antimicrob Agents Chemother. 2021 Jun 17;65(7):e02520-20. doi: 10.1128/AAC.02520-20 (PMC8218629; doi:10.1128/AAC.02520-20)
Supplement: Supplemental file 1 — Supplemental Tables S1 to S3. Download AAC.02520-20-s0001.pdf, PDF file, 0.2 MB [file aac.02520-20-s0001.pdf]

Table S1 Patient treatment history, including phenotypic drug resistance testing, over five TB treatment episodes. Phenotypic drug susceptibility testing (pDST) results to individual drugs were reported by several diagnostic labs throughout Germany including Research Centre Borstel. Drugs that were tested resistant earlier may not have been tested repeatedly, thus the pDST results at given intervals are not completely comprehensive. Emerging genotypic resistance and susceptibility were predicted for 13 available patient derived isolates; date of collection indicated by months since the start of therapy "Therapy (month)".

Therapy (month)	Hospital	Treatment Regimen	Clinical notes	Phenotypic Resistance	Phenotypic Susceptibility	Genotypic Resistance	Active drugs in treatment by genotype
**1st TB Episode**							
1-4	1	H, PAS, S[†], tyloxapol[‡]					
**No TB symptoms - 17 years**							
**2nd TB Episode**							
1	2	H, E, S					
2-4	3	H, R, E	Culture negative				
**No TB symptoms - 1 year**							
**3rd TB Episode**							
1-6	4	H, R, S	Cavities in left and right upper lobes				
7-41	OP	H, R, S	No bacilli detected				
**No TB symptoms - 8 years**							
**4th TB Episode**							
1	-	Z, S, Trd	Bacilli detected and lung cavitation observed				
1-3	5	H, R, E, Z, Cm					
4	5	H, R, E, PAS	Culture positive	(month 4) H, E, Z, Pto, S	(month 4) R, PAS		
5-12	6	Z, S, Trd	Sputum negative	(month 9) H, R	(month 9) E, Z, Pto, S, Cm, Cs, Km		

Table S1 continued							
**Therapy (month)**	**Hospital**	**Treatment Regimen**	**Clinical notes**	**Phenotypic Resistance**	**Phenotypic Susceptibility**	**Genotypic Resistance**	**Active drugs in treatment by genotype**
13-15	-	R, Z, S, Trd	Sputum positive	(month 15) H, R	(month 15) E, S		
16	-	E, Z, S					
16-20	OP	Z, S, Trd					
21-22	OP	R, Z, S, Trd					
23-35	OP	R, Z, S, Trd	Culture positive	(month 24/26/35) H, R	(month 24/26/35) E, S		
36-64	-	R, Z, S, Trd	Culture positive	(month 39) H, R, Pto	(month 39) E, Z, S		
65-88	OP	R, Clr, Pto	Alternating between positive and negative culture and microscopy				
**No TB symptoms - ~6 months**							
**5th TB Episode**							
1	7	Rbt, PAS, Clr, Ofx	Culture positive	H, R, E, Z, S			
2-12	7, OP	Rbt, S, PAS, Clr, Ofx	Culture positive, new infiltration in left and right lungs	(month 10) H, R/Rbt, Z, S, PAS, Ofx, Cfz, Trd/Cs	(month 10) E[b], Cm[b]	(Month 10) Baseline resistance: H, R/Rbt, Z, S, PAS, Pto, Cs/Trd, FQ(Ofx)	1 (Clr*)
13-14	8,9	H, E, Pto, Am, Amx				(Month 13) Baseline	3 (E/Am/Amx*)
15-21	OP	H, E, Pto, Amx	Am discontinued due to hearing loss				2 (Amx*/E)
22-25	OP,9	H, E, Pto, Ofx, Amx	Culture positive	(month 22) H, R, E, Z, Ofx, PAS, Cfz, Cs, Amx	(month 22) Pto, Am, Cm, S[b]		2(Amx*/E)
26-34	OP	H, E, Ofx					1 (E)

Table S1 continued							
**Therapy (month)**	**Hospital**	**Treatment Regimen**	**Clinical notes**	**Phenotypic Resistance**	**Phenotypic Susceptibility**	**Genotypic Resistance**	**Active drugs in treatment by genotype**
35-39	OP	E, Pto, Ofx, Amx					2 (Amx*/E)
40-42	10	H, E, Pto, Ofx, Amx					2 (Amx*/E)
43-44	10	H, E, Z, Pto, Ofx, Amx	Culture positive		(month 43) Z, Pto, PAS, Cm, Cfx (FQ)		2 (Amx*/E)
45-51	OP	H, E, Pto, Ofx, Amx	Culture & microscopy positive	(month 48) H, R, E, Z, S, Clr, Ofx, Mfx, Lfx, Cfz, Cs/Trd	(month 48) Pto, Cm, Am	(month 47) baseline + E	1 (Amx*)
52-80	OP	E, Z, Ofx, Amx	Culture & microscopy positive	(month 71/79) H, R, E, S, Ofx	(month 71) Pto, Cm, Am, Lzd, Z^{b}, Cs/Trd^{b}	(month 71) baseline + E	1 (Amx*)
81	OP	E, Z, Ofx, Amx					1 (Amx*)
82-87	9, 11, OP	Z, Cm, Pto, Lzd	Culture positive, microscopy negative				2 (Cm/Lzd)
88-91	OP	Z, Lzd	Culture positive		(month 89/91) Pto, Cm, Am, Cs/Trd, Lzd, Z^{b}	(month 89) baseline + E	1 (Lzd)
92-93	-	Z, Lzd				(month 92) baseline + E	1 (Lzd)
94	-	Z, Lzd	Culture positive	(month 94) Z, Pto	(month 94) Cm, Am, Lzd, Cs/Trd		1 (Lzd)
95-101	OP	Z, Cm, Pto, Lzd					2 (Cm/Lzd)
102	OP	Lzd, Trd					1 (Lzd)
103-104	OP	Lzd, Trd	Culture positive	(month 104) Pto	(month 104) Z, Cm, Am, Lzd	(month 104) Baseline + E, Lzd/Cm/Clr	0
105-110	OP	Lzd, Trd	Culture positive		(month 108) Z, Cm, Am, Cs/Trd	(Month 108) baseline + E, Lzd	0

Table S1 continued							
**Therapy (month)**	**Hospital**	**Treatment Regimen**	**Clinical notes**	**Phenotypic Resistance**	**Phenotypic Susceptibility**	**Genotypic Resistance**	**Active drugs in treatment by genotype**
111	OP	Lzd, Trd					0
112-129	7, OP	Z, Cm, Trd	Culture positive, Patient quit drinking alcohol	(month 112) Lzd (month 122) Pto	(month 112) Z, Cm, Am, Cs/Trd (month 114) Z Cm, Am (month 122) Cm, Am, Cs/Trd, Z[b]		1 (Cm)
130-132	OP	Z, Cm, Trd	Culture positive		(month 132) Am, Z[b]	(Month 130) baseline + E, Lzd, Cm	0
133	7	Z, PAS, Am, Trd					1 (Am)
134-139	OP	Z, PAS, Am, Trd	Patient quit smoking	(month 134) R, Z, Cm	(month 134) PAS, Am, Cs/Trd	(Month 135) baseline + E, Lzd, Cm	1 (Am)
140	-	Z, PAS, Am, Trd	Culture positive	(month 140) Z, Am	(month 140) Pto, PAS, Cs		1 (Am)
141-153	7	PAS, Pto, Trd	Culture positive	(month 147) Pto	(month 147) PAS, Cs/Trd	(Month 144) baseline + E, Lzd, Cm, Km, Am	0
154-169	OP	NONE	Culture positive	(month 156) R (month 163) PAS	(month 163) Cs/Trd	(Month 160) baseline + E, Lzd, Cm, Km, Am	
170-200	7, OP	Trd	Microscopy positive, radiology indicated progression (month 189)		(month 170) Cs/Trd (month 178) Cs/Trd	(Month 172) baseline + E, Lzd, Cm, Km, Am	0
201	7	Mfx, Trd					0
202-214	OP	-					

215	7	Trd	Microscopy positive				0
216	12						

*Phenotypic test was considered for "Active drugs in treatment" as no genotypic markers are available for these drugs (Clr and Amx)

†Treated with dihydrothenat, a S derivative

‡tyloxapol is an anti-mucosal

[b] border-line resistance (often viewed as some bacterial growth but below or on the boarder of the resistance threshold)

Am: amikacin, Amx: amoxicillin+clavulanic acid, Cfx: ciprofloxacin, Cfz: clofazimine, Clr: clarithromycin, Cm: capreomycin, Cs: cycloserine, E: ethambutol, FQ: fluoroquinolone, H: isoniazid, Km: kanamycin, Lzd: linezolid, Mfx: moxifloxacin, OP: out-patient, PAS: para-aminosalicylic acid, Pto: prothionamide, R: rifampicin, Rbt: rifabutin, S: streptomycin, Sfx: sparfloxacin, Trd: terizidone, Z: pyrazinamide

Table S2 Mutation frequency of drug resistance associated variants of 13 serial bacterial isolates. All resistant variants detected in resistance associated genes, next to correlating drug(s). Frequency of mutation in isolate is organized by column, referencing isolate identification number and date when isolate was collected in month of treatment (of 5th treatment episode). The frequency of each mutation is calculated by the percentage of reads after whole genome sequencing, in which the variant is detected. Only high confidence resistance conferring mutations considered in final analysis, indicated by bold font, literature source included.

				Frequency of mutation in isolate												
			Isolate ID	1060-97	4177-97	2698-00	1633-02	9512-03	31-04	10202-04	3444-05	1126-07	5257-07	3082-08	6974-09	7686-10
			average genome wide coverage	178x	154x	153x	181x	136x	168x	122x	135x	119x	149x	129x	172x	148x
			Month during treatment isolate was collected	10	13	47	71	89	92	104	108	130	135	144	160	172
Drug	Gene Name	Mutation	Literature Reference													
H	*katG*	**S315T**	(1)	100%	100%	100%	99%	100%	99%	100%	100%	100%	99%	100%	100%	100%
	fabG1	-17 g>t	(2)	-	-	-	-	-	-	-	-	-	-	-	-	99%
R/Rbt	*rpoB*	F424V		5%	29.5%	96%	100%	100%	100%	100%	100%	100%	100%	100%	100%	99%
	rpoB	**L430P**	(1)	100%	100%	100%	100%	100%	100%	100%	99%	100%	99%	100%	100%	100%
	rpoB	**D435G**	(1)	99%	100%	100%	99%	99%	99%	100%	100%	100%	100%	99%	100%	99%
E	*embA*	-43 g>c	(2)	99%	99%	99%	100%	100%	99%	100%	100%	99%	99%	99%	99%	100%
	embB	**G406A**	(3)	-	-	93%	99%	100%	100%	100%	100%	100%	100%	99%	100%	99%
Z	*pncA*	**T76P**	(1)	100%	100%	100%	99%	100%	100%	99%	100%	100%	100%	99%	100%	100%
PAS	*ribD*	**-12 g>a**	(4)	99%	99%	100%	99%	100%	100%	99%	100%	100%	99%	100%	100%	100%
S	*gid*	**102 del g**	*(3)	96%	97%	97%	97%	95%	95%	98%	96%	94%	96%	95%	95%	98%
Pto / compen-satory	*Rv0565c*	1312 ins g		-	-	-	-	-	-	97%	-	-	-	-	-	-
	Rv0565c	**C298R**	(5)	-	-	-	-	-	-	-	99%	100%	100%	100%	100%	100%

Table S2 continued																
				Frequency of mutation in isolate												
			Isolate ID	1060-97	4177-97	2698-00	1633-02	9512-03	31-04	10202-04	3444-05	1126-07	5257-07	3082-08	6974-09	7686-10
Month during treatment isolate was collected				10	13	47	71	89	92	104	108	130	135	144	160	172
Drug	Gene Name	Mutation	Literature Reference													
Pto, Eto	*ethA*	**89 del a**		100%	99%	100%	100%	100%	100%	99%	100%	100%	100%	100%	100%	100%
H, Pto, Eto	*Rv3083*	C258F		-	-	-	-	-	-	100%	-	-	-	-	-	-
FQ	*gyrA*	**D94G**	(1)	20.6%	93%	93%	100%	100%	99%	100%	100%	100%	100%	100%	100%	100%
Cm	*tlyA*	**350 ins g**	*(6)	-	-	-	-	-	-	-	-	78.9%	71.4%	97%	96%	99%
	tlyA	**584 ins t**	*(6)	-	-	-	-	-	-	-	-	-	27.5%	-	-	-
Am, Km, Cm	*rrs*	**1401 a>g**	(1)	-	-	-	-	-	-	-	-	-	-	100%	29.5%	99%
Lzd, Cm, Cm	*rrl*	**2746 g>a**	(7)	-	-	-	-	-	-	100%	-	-	-	-	-	-
Lzd	*rplC*	**C154R**	(8)	-	-	-	-	-	-	-	100%	100%	100%	100%	100%	99%
Cfz, Bdq	*Rv0678*	**I67S**	(9)	-	36.4%	-	-	-	-	-	-	-	-	-	-	-
	Rv0678	**R96Q**	*(10)	-	10%	-	-	-	-	-	-	-	-	-	-	-
	Rv0678	**132 ins gt**	*(9)	22.4%	-	3.6%	-	-	-	-	-	-	-	-	-	-
Cs, Trd	*ald*	L343V		-	96%	-	-	-	-	-	-	-	-	-	-	-
	ald	**77 ins a**	*(11)	99%	99%	96%	97%	96%	96%	99%	97%	97%	98%	99%	98%	96%
tolerance associated	*prpR*	**F334L**	*(12)	-	-	-	-	-	100%	-	100%	100%	100%	100%	100%	100%

*Other mutations described in literature reference.

Am: amikacin, Bdq: bedaquiline, Cfz: clofazimine, Clr: clarithromycin, Cm: capreomycin, Cs: cycloserine, E: ethambutol, Eto: ethionamide, FQ: fluoroquinolone, H: isoniazid, Km: kanamycin, Lzd: linezolid, Mfx: moxifloxacin, Ofx: ofloxacin, PAS: para-aminosalicylic acid, Pto: prothionamide, R: rifampicin, Rbt: rifabutin, S: streptomycin, Trd: terizidone, Z: pyrazinamide

Table S3 Statistical analysis of low frequency variants. All low frequency (<75%) mutations were verified using low-frequency SNP detection tool binoSNP. Only high quality calls (minimum base quality: phred = 20) were considered at each position. Variant was included in final dataset if statistically significant ($p \leq 0.05$).

Isolate	POS	Gene	Mutation	REF	ALT	DP	#ALT	Freq (ALT)	p-value	antibiotic
1060-97	7582	*gyrA*	D94G	A	G	155	32	20.6%	7.16E-70	FQ
1060-97	761076	*rpoB*	F424V	T	G	154	7	4.5%	8.29E-12	R
1060-97	779121	*Rv0678*	132 ins gt	G	GT	210	47	22.4%	1.45E-44	Bdq/Cfz
1060-97	3087846	*ald*	L343V	C	G	114	1	0.877%	0.75	Cs/Trd
4177-97	761076	*rpoB*	F424V	T	G	139	41	29.5%	2.68E-94	R
4177-97	779121	*Rv0678*	132 ins gt	G	GT	160	2	1.3%	0.5726	Bdq/Cfz
4177-97	779189	*Rv0678*	I67S	T	G	132	48	36.4%	1.48E-117	Bdq/Cfz
4177-97	779276	*Rv0678*	R96Q	G	A	120	12	10.0%	1.68E-23	Bdq/Cfz
2698-00	779121	*Rv0678*	132 ins gt	G	GT	193	7	3.63%	0.00915	Bdq/Cfz
1126-07	1918523	*tlyA*	584 ins t	-	T	106	2	1.9%	0.363	Cm
5257-07	1918289	*tlyA*	350 ins g	A	G	154	110	71.4%	1.85E-173	Cm
5257-07	1918523	*tlyA*	584 ins t	-	T	131	36	27.5%	4.83E-38	Cm
6974-09	1473246	*rrs*	1401 a>g	A	G	254	75	29.5%	1.28E-178	Am, Km, Cm

ALT: alternative allele, #ALT: number of alternative alleles at position, DP: filtered coverage depth at position, Freq(ALT): frequency of alternative allele, POS: position, REF: reference allele

Am: amikacin, Bdq: bedaquiline, Cfz: clofazimine, Cm: capreomycin, Cs: cycloserine, FQ: fluoroquinolone, Km: kanamycin, R: rifampicin, Trd: terizidone

References

1. Georghiou S, Rodwell T, Colman R, Miotto P, Bainomugisa A, Cabibbe A, Suresh A, Denkinger C, Gilpin C, Korobitsyn A, Dean A, Weyer K, Zignol M. 2018. The use of next-generation sequencing technologies for the detection of mutations associated with drug resistance in Mycobacterium tuberculosis complex: technical guideWHO Global TB Programme.
2. Allix-Béguec C, Arandjelovic I, Bi L, Beckert P, Bonnet M, Bradley P, Cabibbe AM, Cancino-Muñoz I, Caulfield MJ, Chaiprasert A, Cirillo DM, Clifton DA, Comas I, Crook DW, De Filippo MR, de Neeling H, Diel R, Drobniewski FA, Faksri K, Farhat MR, Fleming J, Fowler P, Fowler TA, Gao Q, Gardy J, Gascoyne-Binzi D, Gibertoni-Cruz AL, Gil-Brusola A, Golubchik T, Gonzalo X, Grandjean L, He G, Guthrie JL, Hoosdally S, Hunt M, Iqbal Z, Ismail N, Johnston J, Khanzada FM, Khor CC, Kohl TA, Kong C, Lipworth S, Liu Q, Maphalala G, Martinez E, Mathys V, Merker M, Miotto P, Mistry N, Moore DAJ, Murray M, Niemann S, Ong RTH, Peto TEA, Posey JE, Prammananan T, Pym A, Rodrigues C, Rodrigues M, Rodwell T, Rossolini GM, Padilla ES, Schito M, Shen X, Shendure J, Sintchenko V, Sloutsky A, Smith EG, Snyder M, Soetaert K, Starks AM, Supply P, Suriyapol P, Tahseen S, Tang P, Teo YY, Thuong TNT, Thwaites G, Tortoli E, Omar S V., van Soolingen D, Walker AS, Walker TM, Wilcox M, Wilson DJ, Wyllie D, Yang Y, Zhang H, Zhao Y, Zhu B. 2018. Prediction of susceptibility to first-line tuberculosis drugs by DNA sequencing. N Engl J Med 379:1403–1415.
3. Walker TM, Kohl TA, Omar S V., Hedge J, Del Ojo Elias C, Bradley P, Iqbal Z, Feuerriegel S, Niehaus KE, Wilson DJ, Clifton DA, Kapatai G, Ip CLC, Bowden R, Drobniewski FA, Allix-Béguec C, Gaudin C, Parkhill J, Diel R, Supply P, Crook DW, Smith EG, Walker AS, Ismail N, Niemann S, Peto TEA, Davies J, Crichton C, Acharya M, Madrid-Marquez L, Eyre D, Wyllie D, Golubchik T, Munang M. 2015. Whole-genome sequencing for prediction of Mycobacterium tuberculosis drug susceptibility and

resistance: A retrospective cohort study. Lancet Infect Dis https://doi.org/10.1016/S1473-3099(15)00062-6.

4. Zhang X, Liu L, Zhang Y, Dai G, Huang H, Jina Q. 2015. Genetic determinants involved in p-aminosalicylic acid resistance in clinical isolates from tuberculosis patients in northern China from 2006 to 2012. Antimicrob Agents Chemother.

5. Hicks ND, Carey AF, Yang J, Zhao Y, Fortunea SM. 2019. Bacterial genome-wide association identifies novel factors that contribute to ethionamide and prothionamide susceptibility in mycobacterium tuberculosis. MBio 10.

6. Maus CE, Plikaytis BB, Shinnick TM. 2005. Mutation of tlyA confers capreomycin resistance in Mycobacterium tuberculosis. Antimicrob Agents Chemother 49:571–577.

7. Zhang S, Chen J, Cui P, Shi W, Shi X, Niu H, Chan D, Yew WW, Zhang W, Zhang Y. 2016. Mycobacterium tuberculosis mutations associated with reduced susceptibility to Linezolid. Antimicrob Agents Chemother https://doi.org/10.1128/AAC.02941-15.

8. Beckert P, Hillemann D, Kohl TA, Kalinowski J, Richter E, Niemann S, Feuerriegel S. 2012. rplC T460C identified as a dominant mutation in linezolid-resistant Mycobacterium tuberculosis strains. Antimicrob Agents Chemother 56:2743–2745.

9. Sonnenkalb L, Carter J, Spitaleri A, Iqbal Z, Hunt M, Malone K, Utpatel C, Cirillo DM, Rodrigues C, Nilgiriwala KS, CRyPTIC Consortium, Fowler PW, Merker M, Niemann S. 2021. Deciphering Bedaquiline and Clofazimine Resistance in Tuberculosis: An Evolutionary Medicine Approach. bioRxiv https://doi.org/https://doi.org/10.1101/2021.03.19.436148.

10. Battaglia S, Spitaleri A, Cabibbe AM, Meehan CJ, Utpatel C, Ismail N, Tahseen S, Skrahina A, Alikhanova N, Mostofa Kamal SM, Barbova A, Niemann S, Groenheit R, Dean AS, Zignol M, Rigouts L, Cirillo DM. 2020. Characterization of genomic variants associated with resistance to bedaquiline and delamanid in naive mycobacterium tuberculosis clinical strains. J Clin Microbiol https://doi.org/10.1128/JCM.01304-20.

11. Desjardins CA, Cohen KA, Munsamy V, Abeel T, Maharaj K, Walker BJ, Shea TP, Almeida D V., Manson AL, Salazar A, Padayatchi N, O'Donnell MR, Mlisana KP, Wortman J, Birren BW, Grosset J, Earl AM, Pym AS. 2016. Genomic and functional analyses of Mycobacterium tuberculosis strains implicate ald in D-cycloserine resistance. Nat Genet https://doi.org/10.1038/ng.3548.

12. Hicks ND, Yang J, Zhang X, Zhao B, Grad YH, Liu L, Ou X, Chang Z, Xia H, Zhou Y, Wang S, Dong J, Sun L, Zhu Y, Zhao Y, Jin Q, Fortune SM. 2018. Clinically prevalent mutations in Mycobacterium tuberculosis alter propionate metabolism and mediate multidrug tolerance. Nat Microbiol 3:1032–1042.
